# A survey on robotic devices for upper limb rehabilitation

**DOI:** 10.1186/1743-0003-11-3

**Published:** 2014-01-09

**Authors:** Paweł Maciejasz, Jörg Eschweiler, Kurt Gerlach-Hahn, Arne Jansen-Troy, Steffen Leonhardt

**Affiliations:** 1 DEMAR - LIRMM, INRIA, University of Montpellier 2, CNRS, Montpellier, 161 rue Ada, 34095 Montpellier, France; 2 Institute of Metrology and Biomedical Engineering, Warsaw University of Technology, ul. Św. A. Boboli 8, 02-525 Warszawa, Poland; 3 Helmholtz-Institute for Biomedical Engineering, RWTH Aachen University, Pauwelsstraße 20, 52074 Aachen, Germany; 4 Chair of Medical Engineering (mediTEC), Helmholtz-Institute for Biomedical Engineering, RWTH Aachen University, Pauwelsstraße 20, 52074 Aachen, Germany; 5 Philips Chair of Medical Information Technology (MedIT), Helmholtz-Institute for Biomedical Engineering, RWTH Aachen University, Pauwelsstraße 20, 52074 Aachen, Germany

## Abstract

The existing shortage of therapists and caregivers assisting physically disabled individuals at home is expected to increase and become serious problem in the near future. The patient population needing physical rehabilitation of the upper extremity is also constantly increasing. Robotic devices have the potential to address this problem as noted by the results of recent research studies. However, the availability of these devices in clinical settings is limited, leaving plenty of room for improvement. The purpose of this paper is to document a review of robotic devices for upper limb rehabilitation including those in developing phase in order to provide a comprehensive reference about existing solutions and facilitate the development of new and improved devices. In particular the following issues are discussed: application field, target group, type of assistance, mechanical design, control strategy and clinical evaluation. This paper also includes a comprehensive, tabulated comparison of technical solutions implemented in various systems.

## Introduction

An individual’s capacity to move is necessary to perform basic activities of daily living (ADL). Movement disorders significantly reduce a patient’s quality of life. Disorders of the upper extremities specifically limit the independence of affected subjects. Fortunately, there are various approaches to restore the functionality of the upper extremity, e.g., orthoses, functional electrical stimulation, and physical therapy. Positive outcome of physical rehabilitation, in the case of neurologically based disorders, depends heavily on: onset, duration, intensity and task-orientation of the training [1,2], as well as the patient’s health condition, attention and effort [3]. Intense repetitions of coordinated motor activities constitute a significant burden for the therapists assisting patients. In addition and due to economical reasons, the duration of primary rehabilitation is getting shorter and shorter [4]. These problems will probably exacerbate in the future as life expectancy continues to increase accompanied by the prevalence of both moderate and severe motor disabilities in the elderly population [5] and consequently increasing their need of physical assistance. To counteract these problems, prevailing research studies present a wide variety of devices specifically assisting physical rehabilitation. Robotic devices with the ability to perform repetitive tasks on patients are among these technically advanced devices. In fact, robotic devices are already used in clinical practice as well as clinical evaluation. However, considering the number of devices described in the literature, so far only a few of them have succeeded to target the subject group — for more details see Table 1. Furthermore, it seems that the outcome of the use of devices already in clinical practice is not as positive as expected [3]. New solutions are being considered. Most of the literature reviews on robotic devices for upper extremity rehabilitation (e.g. [6,7]) concentrate on devices that have already undergone clinical evaluation. Gopura and Kiguchi [8] compared the mechanical design of selected robotic devices for upper extremity rehabilitation. However, no other publication presents a summary of different robotic solutions for upper extremity rehabilitation, including those being in the development phase. An assessment of different technical solutions would provide developers of robotic devices for upper limb rehabilitation an evaluation of solutions that have already been considered, and thus learn from successes as well as shortfalls from other research teams. Hence, a comparison of various robotic devices would facilitate the development of new and improved devices for robotic upper limb rehabilitation. The aim of this paper is to summarize existing technical solutions for physical therapy of the upper limb.

**Table 1:** Robotic devices for upper limb rehabilitation

**System name**,**references**	**DOF**	**Supported movements**	**Main control inputs**	**Actuators**	**Type; field of application**	**Stage of development; additional information**
**Systems assisting shoulder movements**						
Kiguchi [114]	2	Shoulder – FE, AA	sEMG	DC motors (x2)	Stationary system (exoskeleton-based); power assistance	C0 study: 1 hs
**Systems assisting elbow movements**						
Cheng [9]	1	Elbow – FE	sEMG	DC motor	Stationary system; physical therapy	CI study: 5 stroke + 5 hs
Cozens [10]	1	Elbow – FE	Joint angle	Electric servo-motor	Stationary system; physical therapy	CI study: 10 stroke + MS
Kiguchi [170]	1	Elbow – FE	sEMG	DC motor	Stationary system (exoskeleton-based); physical therapy	C0 study: 2 hs
*MARIONET*, Sulzer [142]	1	Elbow – FE	Joint angle	AC servomotor (SEA)	Stationary system (end-effector-based); physical therapy	C0 study: 6 hs
Mavroidis [11]	1	Elbow – FE	Force/torque	DC motor	Portable orthosis (continuous passive motion device); physical therapy	Prototype
*MEM-MRB*, Oda [104]	[1]	[Elbow – flexion]	Joint angular velocity, torque	MRF brake	Stationary system; physical therapy	C0 study: 1 hs
*Myomo e100*, Myomo, Inc.; Stein [172]	1	Elbow – FE	sEMG	DC motor (x1)	Portable orthosis; physical therapy	Commercial system (FDA clearance); CI study: 8 cS
Ögce [171]	1	Elbow – FE	sEMG	DC step motor	Wearable shoulder-elbow orthosis; physical therapy	CI study: 2 traumatic brachial plexus injury
Pylatiuk [153]	1	Elbow – FE	sEMG	Hydraulic	Wearable orthosis; physical therapy	First prototype
Rosen [169]	1	Elbow – FE	sEMG	DC motor (x1)	Stationary system (exoskeleton-based); power assistance	C0 study: 1 hs; predecessor of *CADEN-7*
Song [12]	1	Elbow – FE	sEMG	AC servo motor	Stationary system (end-effector-based); physical therapy	CI studies: 8 cS [12], 7 cS [13], 3 cS [14]
Vanderniepen [143]	1	Elbow – FE	Joint angle	Electric motors (x2) (SEA)	Wearable orthosis; orthopedic physical therapy	Prototype
**Systems assisting forearm movements**						
Kung [15]	1	Forearm – PS	Joint angle, torque	AC servomotor (1)	Stationary system; physical therapy	CI study: 7 cS + 8 hs [16]
**Systems assisting wrist movements**						
*ASSIST*, Sasaki [146]	1	Wrist – flexion	Joint angle	Rotary-type pneumatic actuators (x2)	Wearable orthosis; power assistance	C0 study: 5 hs
Colombo [17]	1	Wrist – FE	Torque	Not specified	Stationary system; physical therapy	CII study: 20(8) cS
Hu [18]	1	Wrist – FE	sEMG	Electric motor	Stationary system (end-effector-based); physical therapy	CI study: 15 cS
Loureiro [100]	[1]	[Wrist – FE]	Hand motion (tremor)	MRF brake	Wearable orthosis; tremor suppression	CI study: 1 ET
*PolyJbot*, Song [175]	1	Wrist – FE	sEMG, joint angle and torque	DC servomotor (x1)	Stationary system; physical therapy	CII study: 27(15) cS [19]
**Systems assisting finger(s) movements**						
*Amadeo*, tyromotion GmbH	5	Fingers (each) – FE	End-point position and force	Electric motors	Stationary system (end-effector-based); physical therapy	Commercial system; CI study: 7 aS [20]
Chen [21]	5	Independent linear movement of each finger	Fingers positions and forces, sEMG	DC linear motors (x5)	Stationary system (end-effector-based); physical therapy	C0 study: 1 hs
*CyberGrasp*, CyberGlove Systems LLC; Turner [22]	[5]	[Resistive force to each finger]	Joint angles (*CyberGlove*)	DC motors (x5)	Force-feedback glove; interactions with virtual environment	Commercial system for other applications, used in some clinical studies e.g. [191,192]
Ertas [23]	1	Concurrent FE of 3 joints of a single finger	Joint angles	DC motor (x1)	Finger exoskeleton (underactuated mechanism); tendon physical therapy	C0 study: 4 hs
Fuxiang [24]	4	Index finger– FE (x3), AA	Joint positions and toques	Linear stepping motors	Modular-finger exoskeleton (continuous passive motion device); physical therapy	C0 study: 3 hs
*Gloreha*, Idrogenet srl	5	Independent passive movement of each finger	Fingers positions	Electric motors (x5)	Portable (Gloreha Lite)/Movable (Gloreha Professional) (end-effector-based, cable-driven); physical therapy	Commercial system (CE mark); CII study: 10(5) sS [25], CI studies: 9 stroke + 3 other diseases [26], 4 cS [27]
*Hand of Hope*, Rehab-Robotics Comp. Ltd., Ho [28]	5	Each finger separately - FE	sEMG	DC linear motors (x5)	Portable system (orthosis); physical therapy	Commercial system (CE Mark), CI study: 8 cS
*HandCARE*, Dovat [113]	5	Independent linear movement of each finger (1 at a time)	Fingers positions and forces	DC motor (x1!)	Stationary system (end-effector-based, cable-driven); physical therapy	CI study: 5 cS + 8 hs
*HEXORR*, Schabowsky [29]	2	Thumb – FE, other fingers together – FE	Fingers positions and forces	DC motor (x1), AC motor (x1)	Stationary system (end-effector-based, cable-driven); physical therapy	CI study: 5 cS + 9 hs
*HIFE*, Mali [183]	2	1 finger – FE	End-point position	DC motors	Haptic interface (end-effector-based); physical therapy	Prototype
*InMotion HAND*, previous name *InMotion 5.0*, Interactive Motion Tech., Inc.; Masia [165]	1	All fingers together – GR	Not specified	DC brushless motor	Add-on module for *InMotion ARM*; physical therapy	Commercial system
Kline [30]	1	All fingers together – extension	Joint angles, sEMG	Pneumatic	Wearable glove; physical therapy	CI study: 1 stroke + hs (np)
Lucas [147]	1	Index finger – flexion (passive extension)	sEMG	Pneumatic (x2)	Wearable orthosis; grasp assistance	CI study: 1 SCI
*MR_CHIROD v.2*, Khanicheh [158]	[1]	[All fingers together – GR]	Finger position and torque	ERF brake	Exercising device (handle-like); physical therapy	C0 study: hs (np); fMRI compatible
*MRAGES*, Winter [157]	[5]	[Fingers (each) – FE]	Finger positions and torques	MRF brakes (5)	Force-feedback glove; physical therapy	Prototype
Mulas [31]	2	Thumb – FE, other fingers together – FE	sEMG, pulleys position	DC servo motors (x2)	Wearable orthosis; physical therapy	CI study: 1 sS
Nathan [167]	1	All fingers together – grasp (passive release)	Hand-held trigger, index and thumb fingers joint angles	FES	Wearable orthosis (glove); physical therapy	CI study: 2 stroke + 1 hs
*PowerGrip*, Broaden Horizons, Inc.	1	Thumb, index and middle finger together – GR	Switches or sEMG	DC motor (1)	Wearable orthosis; grasp assistance	Commercial system
*Reha-Digit*, Reha-Stim; Hesse [32]	1	4 fingers (except the thumb) together – FE	None	DC motor	Portable system (rotating handle); physical therapy	Commercial system (CE mark); CII study: 8(4) sS, CI study: 1 cS
Rosati [144]	1	4 fingers (except the thumb) together – FE	Not selected yet	DC motor (SEA)	Wearable orthosis; physical therapy	Design
Rotella [33]	4	Index finger flexion (x2) (passive extension), thumb – flexion, other fingers together – flexion	Not specified	Electric motors	Wearable orthosis; grasp assistance	Design
*Rutgers Master II-ND*, Bouzit [184]	4	Thumb, index, middle, and ring finger – FE	Actuator translation and inclination	Pneumatic (x4)	Force-feedback glove; interactions with virtual environment	Research device; often used for hand therapy (e.g. [185-187])
*Salford Hand Exoskeleton*, Sarakoglou [34]	7	Index, middle, and ring finger – FE (x2), thumb – FE	Joint angles and end-point force	DC motors	Wearable orthosis (exoskeleton); physical therapy	C0 study: hs (np)
Tong [35]	10	Each finger – FE (x2)	sEMG	Electric linear motors (x10)	Portable system (wearable orthosis); physical therapy	CI study: 2 cS
*TU Berlin Finger Exoskeleton*, Wege [36]	4	1 finger – FE (x3), AA	Joint angles	DC motors (x4)	Finger exoskeleton; physical therapy	C0 study: 1 hs
*TU Berlin Hand Exoskeleton*, Fleischer [117]	20	FE and AA of all major joints of each finger	Joint angles, end-point force, sEMG	DC motors	Wearable orthosis (exoskeleton); physical therapy	Prototype
Worsnopp [37]	3	Index finger – FE (x3)	Joint angles and torques	DC brushless servomotors (x6)	Finger exoskeleton; physical therapy	Prototype
Xing [38]	2	Thumb – FE, other fingers together – FE	Position, force	Pneumatic (PAMs) (x2)	Wearable orthosis; physical therapy	C0 study: 3 hs
**Systems assisting shoulder and elbow movements**						
*ACRE*, Schoone [108]	5	Shoulder * elbow	Joint angles	Electrical motors (x5)	Stationary system (end-effector-based); physical therapy	CI: 10 sS
*ACT*^*3D*^, Ellis [39]	3	Shoulder * elbow	End-point torque, position and velocity (*HapticMaster*)	DC brushed motors (*HapticMaster*)	Stationary system (end-effector-based); physical therapy and assessment of therapy results	CI study: 6 stroke
*ARC-MIME*, Lum [137]	1+[2]	Shoulder * elbow (longitudinal movements of the forearm) [forearm’s elevation and yaw]	Forearm position and torque	DC motor (x1), magnetic particle brakes (x2)	Stationary system (end-effector-based); physical therapy	An attempt to commercialize; CI study: 4 cS; merges concepts from *MIME* and *ARM Guide*
*ARM Guide*, Reinkensmeyer [136]	1+[2]	Shoulder * elbow (longitudinal movements of the forearm) [forearm’s elevation and yaw]	Forearm position and torque	DC motor (x1), magnetic particle brakes (x2)	Stationary system (end-effector-based); physical therapy	CII study: 19(10) cS [40]; see also: *ARC-MIME*
*BFIAMT*, Chang [41]	2	Shoulder * elbow (bilateral longitudinal movements of the forearms)	End point position and torque	DC servomotor (x2), magnetic particle brakes (x2)	Stationary system (end-effector-based); physical therapy	CI study: 20 cS [41]
*BONES*, Klein [118]	4	Shoulder – FE, AA, RT, elbow – FE	Joint angles, cylinder pressure	Pneumatic (x5)	Stationary system (parallel robot + exoskeleton-based distal part); physical therapy	Prototype; see also: *Supinator extender (SUE)*
*Dampace*, Stienen [154]	[4]	[Shoulder – FE, AA, RT, elbow – FE]	Joint angles and torques	Hydraulic brake actuators (SEA)	Stationary system (exoskeleton-based); physical therapy	CI study: stroke (np); see also *Limpact*
Freeman [163]	2	Shoulder * elbow (in the plane)	Handle torque and position	DC brusheless servomotors (x2), FES	Stationary system (end-effector-based); physical therapy	C0 study: 18 hs
*InMotion ARM*, previous name *InMotion 2.0*, Interactive Motion Tech., Inc.; based on: *MIT Manus*, Krebs [107]	2+[1]	Shoulder * elbow (in the plane + gravity compensation)	Joint positions, angular velocity and torque	DC brushless motors	Stationary system (end-effector-based); physical therapy	Commercial system, CIII/CIV studies: 127(49) cS [203], CII studies: 56(30) aS [42], 30(10) aS [43] and others
Ju [44]	2	Shoulder * elbow (in the plane)	Handle torque and position	AC motors (x2)	Stationary system (end-effector-based; physical therapy	CI study: stroke (np)
Kiguchi [45]	3	Shoulder – FE, AA, elbow – FE	sEMG	DC motors	Wheelchair mounted system (exoskeleton-based); power assistance	C0 study: hs (np); see also: shoulder, elbow and shoulder-elbow-forearm orthoses developed by Kiguchi and *SUEFUL-7*
Kobayashi [149]	4	Shoulder – FE, AA, RT, elbow – FE	Joint angle	Pneumatic (PAMs) (x10)	Wearable (but not portable) orthosis (”muscle suit“); power assistance	C0 study: 5 hs
*Limpact*, Stienen [155]	4	Shoulder – FE, AA, RT, elbow – FE	Joint angles and torques	Rotational hydroelastic actuator (SEA)	Stationary system (exoskeleton-based); physical therapy	Design; based on *Dampace*
*MariBot*, Rosati [46]	5	Shoulder * elbow	Motor positions	DC frameless brushless motors	Stationary system (end-effector-based, cable-driven robot); physical therapy	Prototype; successor of *NeReBot*
*MEMOS*, Micera [132]	2	Shoulder * elbow (in the plane)	Torque and handle position	DC motors (x2)	Stationary system (end-effector-based); physical therapy	CII study: 20(12) cS [17], CI study: 18 cS [47]
*MIME*, Burgar [120]	6	Shoulder * elbow	Forearm position, orientation, torque	DC brushed servomotors (*PUMA 560* robot)	Stationary system (end-effector-based); physical therapy	CII studies: 27(13) cS [48] and 30(24) sS [49], CI study: 13 cS [50]; see also *ARC-MIME*
Moubarak [51]	4	Shoulder – FE, AA, RT, elbow – FE	Joint position, velocity and torques	DC brushless motors (x4)	Wheelchair-mounted system (exoskeleton-based); physical therapy	Prototype
*NeReBot*, Rosati [111]	3	Shoulder * elbow	Motor positions	DC motors (x3)	Stationary system (end-effector-based, cable-driven robot); physical therapy	CII studies: 24(12) sS [111], 35 (17) aS [52], 21(11) sS [53]; predecessor of *MariBot*
*REHAROB*, Toth [125]	12	Shoulder * elbow	End-point torques	Electrical motors (*ABB IRB 140* and *IRB 1400H* robots)	Stationary system (2 modified industrial robots); physical therapy	CII study: 22 (13) stroke + 8(2) TBI [54], CI study: 6 cS + 2 sS + 4 hs [125]
**Systems assisting forearm and wrist movements**						
*Bi-Manu-Track*, Reha-Stim; Hesse [55]	1	Forearm – PS * wrist – FE	Not specified	Not specified	Stationary system (end-effector-based); physical therapy	Commercial system, CII study: 44 (22) sA [56], CI study: 12 cS [55]
*CRAMER*, Spencer [109]	3	Forearm – PS, wrist – FE, AA	Hand accelerations (Nintendo Wii console)	Digital servomotors (x4)	Stationary system (parallel robot); physical therapy	Prototype
*InMotion WRIST*, previous name *InMotion 3.0*, Interactive Motion Tech., Inc.; Krebs [138]	3	Forearm – PS, wrist – FE * AA	Joint angles	DC brushless motors (x3)	Stationary system, may be used as an add-on for *InMotion ARM*; physical therapy	Commercial system
*RiceWrist*, Gupta [119]	4	Forearm – PS, wrist – FE * AA	Joint angles and forces	Frameless DC brushless motors	Wearable orthosis; physical therapy	Prototype; extension for *MIME*, see also: *MAHI*
*Supinator extender (SUE)*, Allington [57]	2	Forearm – PS, wrist – FE	Joint angles and forces	Pneumatic	Wearable orthosis; physical therapy	CI study: 8 cS; extension for *BONES* and *ArmeoSpring*
Takaiwa [110]	3	Forearm – PS, wrist – FE, AA	Torque	Pneumatic (x6)	Stationary system (parallel robot); physical therapy	Prototype
*W-EXOS*, Gopura [174]	3	Forearm – PS, wrist – FE, AA	sEMG, hand force, forearm torque	DC motors (x3)	Stationary system (exoskeleton-based); power assistance	C0 study: 2hs; see also: SUEFUL-7
**Systems assisting wrist and fingers movements**						
*AMES*, Cordo [58]	1	wrist and MCP joints of 4 fingers (coupled together)	Flexion/Extension torque, sEMG (optional)	Electric motor + 2 vibrators (for flexor and extensor tendons)	Stationary system (with desktop mounted orthosis), physical therapy (at home)	FDA clearance; CI study: 20(11) cS; a modified version of the system is used for ankle rehabilitation
*Hand Mentor™*, Kinematic Muscles, Inc.; Koeneman [59]	1	Wrist and 4 fingers (except the thumb) extension	Wrist angle, flexion torque	Pneumatic (PAM) (x1)	Wearable orthosis; physical therapy	Commercial system (FDA Class I Device); CII study: 21(11) sS [60], CI studies: 1 cS [61], 1 cS [62]
*HWARD*, Takahashi [130]	3	Wrist – FE, thumb – FE, other fingers together – FE	Joint angles and torques	Pneumatic (x3)	Stationary system (with desktop mounted orthosis); physical therapy	CII study: 13(13) cS
*My Scrivener*, Obslap Reseach, LLC; Palsbo [190]	3	Wrist * fingers	End-point position and torque (*Novint Falcon*)	Electric motors (*Novint Falcon*)	Stationary system (end-effector-based, using haptic device); fine motor hand therapy	CI study: 18 children with weak handwriting skills
**Systems assisting shoulder, elbow and forearm movements**						
*ADLER*, Johnson [63]	3+{3}	Shoulder * elbow * forearm	End-point torque, position and velocity (*HapticMaster*)	DC brushed motors (*HapticMaster*)	Stationary system (end-effector-based); physical therapy	C0 study: 8 hs [64]
*ARAMIS*, Pignolo [65]	6x2	Shoulder – FE, AA, RT, elbow – FE, forearm – PS	Joint angles and torques	DC brushed motors (x6 per exoskeleton)	Stationary system (2 exoskeletons); physical therapy	CI study: 14 sS
*Gentle/S*, Amirabdollahian [121]	3+{3}	Shoulder * elbow * forearm	End-point torque, position and velocity (*HapticMaster*)	DC brushed motors (*HapticMaster*)	Stationary system (end-effector-based); physical therapy	CII study: 31(31) sS + cS [66]; predecessor of *Gentle/G*
*iPAM*, Culmer [67]	6	Shoulder * elbow * forearm	Joint torques	Pneumatic	Stationary system (2 robotic arms); physical therapy	CI study: 16 cS
Kiguchi [68]	4	Shoulder – FE, AA, elbow – FE, forearm – AA	sEMG	DC motors	Wheelchair mounted system (exoskeleton-based); power assistance	C0 study: 1 hs; see also: shoulder, elbow and shoulder-elbow orthoses developed by Kiguchi and *SUEFUL-7*
*L-Exos*, Frisoli [197]	4	Shoulder – FE, AA, RT, elbow – FE {forearm – PS}	Joint angles	Electric motors (x4)	Stationary system (exoskeleton-based); physical therapy	CI study: 9 cS [69]
*MGA*, Carignan [70]	5	Shoulder – FE, AA, RT, VD, elbow – FE, {forearm – PS}	Joint torques	DC brushless motors (x5)	Stationary system (exoskeleton-based); physical therapy	Prototype
*MULOS*, Johnson [168]	5	Shoulder – FE, AA, RT, elbow – FE, forearm – PS	Joystick (4 DOF)	Electric motors (x5)	Wheelchair-mounted system (exoskeleton-based); power assistance and physical therapy	C0 study: 1 hs
*NJIT-RAVR*, Fluet [71]	3+{3}	Shoulder * elbow * forearm	End-point torque, position and velocity (*HapticMaster*)	DC brushed motors (*HapticMaster*)	Stationary system (end-effector-based); physical therapy of children	CI study: 8 CP
*RehabExos*, Vertechy [131]	4	Shoulder – FE, AA, RT, elbow – FE {forearm – PS}	Joint torques	Custom-made frameless brushless motor (x3), DC motor (x1)	Stationary system (exoskeleton-based); physical therapy	First prototype
**Systems assisting shoulder, elbow and fingers movements**						
*Pneu-WREX*, Wolbrecht [145]	4+{1}	Shoulder – FE, AA, HD, elbow – FE, {fingers – GR}	Joint angles, grasp force, cylinder pressure	Pneumatic (x4)	Stationary system (exoskeleton-based); physical therapy	CI study: 11 cS [72]; see also: *T-WREX* and *ArmeoSpring*
*T-WREX*, Sanchez [106]	{5}	{Shoulder – FE, AA, RT, elbow – FE, fingers – GR}	Joint angles, grasp force	None	Wheelchair mounted gravity balancing orthosis; physical therapy	CII studies: 23(11) cS [73], 28(14) cS [74], CI studies: 9cS + 5cS (2 studies) [75]; see also: *Pneu-WREX* and *ArmeoSpring*
**Systems assisting elbow, forearm and wrist movements**						
Ding [179]	4	Elbow – FE, forearm – PS, wrist – FE, AA	Joint angles (a Motion Capture System is used)	Pneumatic (x8)	Wearable (but not portable) orthosis; power assistance for explicitly specified muscles	C0 study: 6 hs
*MAHI*, Gupta [76]	5	Elbow – FE, forearm – PS, wrist – FE * AA	Joint angles	Frameless DC brushless motors	Wearable orthosis (force-feedback exoskeleton); physical therapy	Prototype; extension for *MIME*; see also: *RiceWrist*
*WOTAS*, Rocon [99]	[3]	[Elbow – FE, forearm – PS, wrist – FE]	Angular velocity, torques	DC motors (x3)	Wearable orthosis; tremor suppression	CI study: 10 mainly ET
**Systems assisting forearm, wrist and fingers movements**						
*Haptic Knob*, Lambercy [77]	2	Forearm – PS * wrist – FE, fingers – GR	Position, torque	DC brushed motors (x2)	Stationary system (2 parallelograms); physical therapy	CI study: 3 cS
Hasegawa [98]	11	Forearm – PS, wrist – FE, AA, thumb – FE (x2), index finger – FE (x3), other fingers together –FE (x3)	sEMG	DC motors (x11)	Wearable orthosis; grasp assistance	C0 study: 1 hs
Kawasaki [178]	18	Forearm – PS, wrist – FE, thumb – FE (x3), AA, other fingers – FE (x2), AA	Joint angles of healthy hand	Servo motors (x22)	Stationary system (exoskeleton-based); physical therapy	C0 study: 1 hs
Scherer [156]	[1]	[Forearm and fingers twisting movements * wrist – FE]	Position, torque	Magnetic particle brake	Stationary system (end-effector-based, rotating handle); physical therapy	CI study: 2 stroke + 1 MS
**Systems assisting shoulder, elbow, forearm and wrist movements**						
*Braccio di Ferro*, Casadio [134]	2	Shoulder * elbow * (forearm) * wrist (in the horizonatal or vertical plane)	Device joint angles, end-point force	AC brushless servomotors (x2)	Stationary system (end-effector-based); physical therapy	CI studies: 10 cS + 4 hs [78], 7 MS + 9 hs [79], 11 MS + 11 hs [80], 8 MS [81]
*CADEN-7*, Perry [97]	2x7	Shoulder – FE, AA, RT, elbow – FE, forearm – PS, wrist – FE, AA	sEMG, joint angles, angular velocities and forces/torques	DC brushed motors (2x7)	Stationary system (exoskeleton-based), 2 robotic arms; power assistance	C0 study: 1 hs
Denève [82]	3	Shoulder * elbow * (forearm) * wrist	Device joint angles, end-point force	AC brushless motors (x3)	Stationary system (end-effector-based); physical therapy	Prototype
*EMUL*, Furusho [159]	3	Shoulder * elbow * (forearm) * wrist	End-point position	Electric motors + ERF clutches	Stationary system (end-effector-based); physical therapy	CI study: 6 stroke; predecessor of *PLEMO*, see also: *Robotherapist*
*ESTEC exoskeleton*, Schiele [115]	9	Shoulder – FE, AA, RT, VD, HD, elbow – FE, forearm – PS, wrist – FE, AA	Joint angles	Not selected yet	Wearable system (exoskeleton-based); physical therapy	First prototype
Furuhashi [83]	3	Shoulder * elbow * (forearm) * wrist	End-point torque	DC motors (x3)	Stationary system (end-effector-based); physical therapy	Prototype
*Hybrid-PLEMO*, Kikuchi [135]	2	Shoulder * elbow * (forearm) * wrist (in the adjustable plane)	Device joint angles, end-point force	DC servomotors (x2) + ERF clutches/brakes (x4)	Stationary system (end-effector-based); physical therapy	Prototype; based on *PLEMO*
Lam [180]	2	Shoulder * elbow * (forearm) * wrist (in the plane)	End-point position, abnormal trunk position detection	Not specified	Stationary system (end-effector-based); physical therapy	C0 study: 8 hs
Li [176]	5	Shoulder – FE, AA, elbow – FE, forearm – PS, wrist – FE	sEMG signals from not affected arm	AC (x3) and DC (x2) servo motors	Wearable system (exoskeleton-based); physical therapy	Prototype
*MACARM*, Beer [112]	6	Shoulder * elbow * forearm * wrist	End-point position and force	Electric motors (x8)	Stationary system (end-effector-based, cable-driven robot); physical therapy	CI study: 5 cS
Mathai [84]	3	Shoulder * elbow * forearm * wrist	End-point torque, position and velocity (*HapticMaster*)	DC brushed motors (*HapticMaster*)	Stationary system (end-effector-based); physical therapy	CI study: 4 cS
*MIME-RiceWrist*, Gupta [119]	10	Shoulder * elbow * forearm * wrist	See separate information for MIME and RiceWrist system	See separate information for MIME and RiceWrist system	Stationary system (robotic arm + orthosis); physical therapy	CI study: stroke (np)
*PLEMO*, Kikuchi [105]	[2]	[Shoulder * elbow * (forearm) * wrist] (in the adjustable plane)	Device joint angles, end-point force	ERF brakes	Stationary system (end-effector-based); physical therapy	CI study: 6 stroke + 27 hs [85]; successor of *EMUL*, predecessor of Hybrid-PLEMO
*Robotherapist*, Furusho [160]	6	Shoulder * elbow * forearm * wrist	End-point position	Electric motors + ERF clutches	Stationary system (end-effector-based); physical therapy	Prototype; see also: *EMUL*
*RUPERT IV*, Balasubrama- nian [151]	5	Shoulder – AA, RT, elbow – FE, forearm – PS, wrist – FE	Joint torques and actuators pressure	Pneumatic (PAMs)	Wearable system (exoskeleton-based); physical therapy	CI study: 6 cS [86]
*Salford Arm Rehabilitation Exoskeleton*, Tsagarakis [148]	7	Shoulder – FE, AA, RT, elbow – FE, forearm – PS, wrist – FE, AA	Joint positions and torques	Linear pneumatic actuators (PAMs) (x14)	Stationary system (exoskeleton-based); physical therapy	Prototype
*Sophia-3*, Rosati [87]	2	Shoulder * elbow * (forearm) * wrist (in the plane)	End-point position and force	AC motors	Stationary system (end-effector-based, planar cable-driven robot); physical therapy	First prototype; see also: *Sophia-4*
*Sophia-4*, Rosati [87]	2	Shoulder * elbow * (forearm) * wrist (in the plane)	End-point position and force	DC motors	Stationary system (end-effector-based, planar cable-driven robot); physical therapy	Prototype; see also: *Sophia-3*
*SUEFUL-7*, Gopura [166]	7	Shoulder – FE, AA, RT, elbow – FE, forearm – PS, wrist – FE, AA	sEMG/joint forces/torques	DC servo motors (x7)	Stationary system (exoskeleton-based); power assistance	C0 study: 2 hs; shoulder-elbow orthosis integrated with *W-EXOS* system
Takahashi [182]	2	Shoulder * elbow * (forearm) * wrist (in the plane)	End point position	Electric servomotors (x2)	Stationary system (end-effector-based); physical therapy	CI study: 5 stroke + 2 Guillain-Bare syndrome
Tanaka [88]	2	Shoulder * elbow * (forearm) * wrist (in the plane)	End-point force and position	AC linear motor (x2)	Stationary system (end-effector-based); physical therapy	C0 study: 6 hs
*UHD*, Oblak [139]	2	3 configurations possible: 1) shoulder * elbow, 2) forearm – PS, wrist – FE, 3) forearm – PS, wrist – AA	Torque and handle position	DC motors (x2), (SEA)	Stationary system (end-effector-based); physical therapy	CI study: 1 cS; reconfigurable robot
Umemura [152]	7	Shoulder – FE, AA, RT, elbow – FE, forearm – PS, wrist – FE, AA	Actuators pressure	Hydraulic	Stationary system (end-effector-based); physical therapy	Prototype
*UMH*, Morales [127]	6	Shoulder * elbow * forearm * wrist	Joint torques	Pneumatic	Stationary system (two robotic arms); physical therapy	C0 study: hs (np)
Xiu-Feng [89]	2	Shoulder * elbow * (forearm) * wrist (in the plane)	Device joint angles, end-point force	AC servomotors (x2)	Stationary system (end-effector-based); physical therapy	CI study: 30 stroke
**Systems assisting shoulder, elbow, forearm, wrist and finger movements (whole arm)**						
*ArmeoPower*, Hocoma AG; based on: *ARMin III*, Nef [90]	6{+1}	Shoulder – FE, AA, RT, elbow – FE, forearm – PS, wrist – FE, {fingers – GR}	Joint angles, grasp force	DC motors (x6)	Stationary system (exoskeleton-based); physical therapy	Commercial system; CI studies: 3 cS (ARMin I) [91], 4 cS (ARMin II) [92]
*ArmeoSpring*, Hocoma AG; based on: *T-WREX*, Sanchez [106]	{7}	{Shoulder – FE, AA, RT, elbow – FE, forearm – PS, wrist – FE, fingers – GR}	Joint angles, grasp force	None	Stationary system (exoskeleton-based); physical therapy	Commercial system (CE Mark, FDA clearance); CI study: 10 MS [93]; see also: *T-WREX*
*ARMOR*, Mayr [177]	8	Shoulder – FE, AA, RT, elbow – FE, forearm – PS, wrist – FE, thumb – FE, other fingers together – FE	Joint angles of the master hand	Electric motor	Stationary master-slave system (exoskeleton-based); physical therapy	CII study: 8(8) sS
*Gentle/G*, Loureiro [123]	6{+3}	Shoulder * elbow (3 DOF, *HapticMaster*), {forearm – PS, wrist – FE, AA}, thumb – FE, other fingers together – FE (x2) (grasp robot)	End-point torque, position and velocity (*HapticMaster*) joint angels and end-point force (grasp robot)	DC brushed motors (*HapticMaster* and grasp robot)	Stationary system (robotic arm + orthosis); physical therapy	CII study: 4(4) sS [94]; based on *Gentle/S*
*HEnRiE*, Mihelj [124]	4{+2}	Shoulder * elbow (3 DOF, *HapticMaster*), {wrist – FE, AA}, thumb, middle and index finger together – GR	End-point torque, position and velocity (*HapticMaster*) joint angels and end-point force	DC brushed motors (*HapticMaster*) electric motors (grasping device)	Stationary system (robotic arm + orthosis); physical therapy	Prototype (with spring instead of an actuator in the hand part); C0 study: 1 hs; based on *Gentle/S*
*IntelliArm*, Ren [116]	8{+2}	Shoulder – FE, AA, RT, VD, {HD (x2)}, elbow – FE, forearm – PS, wrist – FE, all fingers together – GR	Joint angles and torques	Not specified	Stationary system (exoskeleton-based); physical therapy	CI study: stroke (np)
*MUNDUS*, Pedrocchi [101]	[3]+{2}+1	[Shoulder – FE, AA, elbow – FE], optional: forearm – PS, wrist – FE (shoulder-elbow-wrist exoskeleton), optional: all fingers together – GR (hand orthosis)	sEMG, button, eye-movement or Bran Computer Interface; object labels – radio frequency identification	elastic elements or DC brakes (shoulder-elbow-wrist exoskeleton), FES (optional), DC motor (optional hand orthosis)	Modular wheelchair-mounted system (exoskeleton-based); movement assistance	CI study: 3 SCI + 2 MS
*ReoGo*, Motorica Medical Inc.	2+{1}	Shoulder * elbow; also {* wrist} or {fingers - FE} if special handle used	End-point position	Electric motors (x4)	Portable system (end-effector-based) with various handles; physical therapy	Commercial system; CIII/CIV study: 60(np) sS [198], CI studies: 14 cS [95], 10 sS [96]

All the systems in the following table are grouped according to the joint movement they support. For the sake of convenience, we consider the shoulder complex, the forearm and the hand (fingers) as single joints. Thus, we distinguish the following “joints”: shoulder, elbow, forearm, wrist and fingers. Devices assisting movements of only one “joint” (starting from shoulder and ending with fingers) are described first followed by devices assisting movement of two, three and four joints (in that order). The end of the table presents systems assisting movement of the whole arm.

For some systems it was difficult to classify them into a particular group. One of such cases includes the end-effector-based systems with a splint. A specific classification to particular group may depend on the joints constrained in particular case by the splint. Furthermore, some devices allow for movements in some joints only in a limited range.

In some cases the same system may appear multiple times in the table on various stages of development. We have accepted such occurrences only if, in our opinion, the difference between two versions of the system justified considering them as two various systems. Otherwise, information included in the table includes only the most recent version of the system available at the time of this publication.

System names are provided in italics. Whenever possible, the first column of the table provides the system name and reference (including the name of the first author) to the publication in which the system is described. We only provide the appropriate reference for systems without a name. The names of commercial systems are followed by their producer names. Appropriate information is provided following a semicolon for commercial systems based on systems being described in scientific publication before commercialization. Except one case, i.e. *ArmeoSpring* based on *T-WREX* system, the description of the predecessors is not provided elsewhere in the table because we found no significant differences between the predecessors and their commercial versions.

The last column contains information about the current stage of system development, clinical trials performed using the system and some additional information are provided. If the system has undergone clinical evaluation, information about the category of the trial, number of participants enrolled and their condition, as well as reference to the paper presenting results of the study is also provided. We distinguish four categories of the studies marked as C0, CI, CII, CIII/CIV. For a description, see Table 7. Categories CII and CIII/CIV provide two numbers of subjects. The first number indicates the total number of participants enrolled in the study. The number in parenthesis indicates number of participants undergoing therapy using the particular system. We made this distinction because there is often a control group undergoing other form of therapy in the CII and CIII/CIV studies. If both numbers are equal, all participants underwent therapy using the specified system but other parameter of the study varied between the groups (e.g. training intensity, device control strategy, or order in which various forms of therapy were applied). No reference after the number and condition of participants indicates that the reference is the same as the one provided in the first column. Information about predecessors or successors is also provided, if available. We use the following symbols and abbreviations: - for degrees of freedom of the device (DOF) and supported movements (second and third column of the table respectively): [ ] - indicates passive (i.e. exerting only resistive force) and { } - indicates not-actuated degrees of freedom or movements, otherwise active. - for supported movements (third column): (joint name) - indicates that range of movements for that joint is limited to a very small range, AA – adduction/abduction, FE – flexion/extension, GR – grasp and release, PS – pronation/supination, RT – internal/external rotation, HD - horizonatal displacement, VD - vertical displacement (both in the shoulder girdle), MCP – metacarpophalangeal joint, * - indicates that the direction of the movement of the device does not correspond to the direction of any of basic anatomical movements (e.g. pronation/supination, flexion/extension, rotation) but is a combination of many, (x number) - indicates that a few particular movements are possible (e.g. flexion in a few joints of one finger), (in the plane) - indicates that the end effector of the device moves only in a specified plane; for the explanation of anatomical terms of motion see Figure 2. - for main control inputs and actuators (fourth and fifth column respectively): (*commercial system name*) - indicates that the particular commercial device (usually robot or haptic interface) is incorporated in the described system and that the particular sensors or actuators are part of that commercial system. - for main control (forth column): sEMG - surface electromyography. - for actuators (fifth column): AC - alternating current, DC - direct current, ERF - electrorheological fluid based, FES - functional electrical stimulation, MRF - magnetorheological fluid based, PAM - pneumatic artificial muscle, SEA - series elastic actuator, (x number) - number of particular actuators being used (provided only if such an information was available). - for clinical studies (last column): C0, CI, CII, CIII/CIV - category of the study: 0, I, II and III/IV, respectively (for category descriptions see the subsection *Clinical studies* of the survey); subject condition: aS - acute stroke, CP - cerebral palsy, cS - chronic stroke, ET - essential tremor, hs - healthy subject(s), MS - multiple sclerosis, SCI - spinal cord injury, sS - subacute stroke, TBI - traumatic brain injury; np - number of subjects is not provided.

The survey of robotic devices is comprised of advanced technology systems. As defined in this report, the design of advance technology systems includes sensors, actuators, and control units; purely mechanical solutions are excluded from this survey. Although the research team made an effort to identify as many systems as possible, it is reasonable to acknowledge that many systems are left unmentioned. Nevertheless, this documentation is intended to be a valuable source of information for engineers, scientists and physiotherapists working on new solutions for physical rehabilitation.

## The survey

### Scope of the survey

At the outset, the research team identified literature associated with the subject matter based on searches in PubMed, the Institute of Electrical and Electronics Engineers (IEEE), Science Direct and Google Scholar databases using various combinations of the following keywords: upper extremity, arm, hand, rehabilitation, therapy, training, movement, motion, assistance, support, robot, robotized, robotic, mechatronic, and motorized. Additionally, referenced literature from the selected publications was included in the survey as well. The information obtained from this literature compendium is supplemented with the data acquired from professional caregivers and manufacturers’ catalogs and websites, as well as direct communications with rehabilitation professionals, manufacturers and patients. Over 120 systems are summarized and compared in Table 1; this tabulated summary constitutes the reference for information provided in subsequent sections. As previously mentioned, the scope of this report is generally limited to the devices that support or retrain movement or manipulation abilities of disabled individuals. This survey excludes systems developed for movement assessment, occupational purposes or boosting physical abilities of healthy people. We however considered some academic, not yet specialized systems, supporting upper-extremity movements, especially if they have potential to be used for rehabilitation purposes (e.g. *CADEN-7*[97]). This survey also excludes devices that substitute movements of the disabled extremity but do not replace the movement itself (e.g. wheelchair mounted manipulators or autonomous robots). Although these devices improve the patient’s quality of life, they differ significantly from systems described in this survey and constitute a separate category of devices. Some companies (e.g. CSMi Computer Sports Medicine, Inc.; Biodex Medical Systems, Inc.; BTE Technologies, Inc.) manufacture sensorized equipment for rehabilitation of various joints and muscles and whose principle of operation often resembles that of exercising devices found at fitness centers. Those devices are used mainly to strengthen muscles and joints and provide some predefined resistance (e.g. isotonic, isometric or isokinetic exercises) or active force (e.g. continuous passive motion exercises). These devices also constitute a different category from the systems included in this survey because their functions are performed along a predefined operation pathway. Although difficult to clearly identify, the aforementioned were also excluded from this review.

Throughout this report, the term “number of degrees of freedom (DOF)” describes the sum of all independent movements (i.e. displacements or rotations) that can be performed in all the joints of the device. The number of DOF is defined in order to determine the exact position and orientation of all segments of the device. Also, some sections in this report are supplemented by an explanation of the most important terminology for readers who are not familiar with the technical vocabulary.

### Application field and target group

A description of the specific field of application for upper limb rehabilitation devices often determines solutions for which the device itself may be applied. Upper-extremity rehabilitation involves actions that stimulate patients’ independence and quality of life. Two main application fields of robotic devices stand out: support to perform some ADLs (e.g. by power assistance or tremor suppression) and providing physical training (therapy). Although there is a significant need for **powered devices supporting basic ADL at home**, there are only a few of such devices proposed so far (see sixth column in Table 1). This is mainly due to technical and economical restrictions. Such devices should significantly improve the lives of their users, otherwise patients become dissatisfied and discontinue their use shortly after. They should be also safe, easily to handle and inexpensive. Portability is also often expected from devices assisting patients to perform basic ADL; in such cases the amount of available energy is limited by the capacity to store energy. Furthermore, if the device is supposed to support movements of multiple joints, the number of needed actuators increases as well as the weight of the device. Therefore, the number of portable actuated devices supporting upper extremity movements is typically low. Instead, purely mechanical solutions are used for that purpose. A few examples of portable powered devices for upper extremity assistance used in daily living are *PowerGrip* system (Broaden Horizons, Inc., USA) and a system proposed by Hasegawa, et al. [98] (both for grasp assistance), as well as *WOTAS* orthosis [99] and a system proposed by Loureiro, et al. [100] (both for tremor suppression). However, portability is not always necessary. Often, especially after a stroke or a spinal cord injury, disorders of the upper extremity are accompanied by lower extremity disabilities. These scenarios are typically characterized by immobilized conditions and require a wheelchair. Therefore, many systems assisting upper limb movements are installed close to the patient (e.g. modular wheelchair-mounted system *MUNDUS*[101]).

Another group of the robotic devices used for rehabilitation purposes, much bigger than the group of devices supporting basic ADLs, constitute **devices providing physical therapy**. These may be designed for either specialized therapeutic institutes or home-based conditions. A vast majority of these devices may be used only at therapeutic institutes since they require supervised assistance from qualified personnel. Their price is often prohibitive for personal use due to their complexity. The patient demand for home-based therapy is expected to increase. Along this context, the concept of the *Gloreha* system (Idrogenet srl) is provided in two versions: (1) a more complex and more adaptable professional version intended for use at hospitals and rehabilitation centers and (2) a simplified low-cost version intended for patient use at home. However, according to Dijkers, et al. [102], many therapists may stop using devices if set-up takes more than 5 minutes. Thus new developed devices for physical training should be intuitive, easy and fast to set-up and have a reasonable price.

Stroke is the most common cause among diseases and injuries for upper limb movement disorders. It is estimated that by 2030, stroke will be the fourth leading cause of reduced disability-adjusted life-years (DALY) in western countries. DALY takes into account years of life lost due to premature death as wells as years of life lived in less than full health [103]. Other causes include traumatic brain injury, spinal cord injury and injuries to motoneurons, as well as certain neurological diseases such as multiple sclerosis, cerebral palsy, Guillain-Barre syndrome, essential tremor and Parkinson’s disease. Currently proposed robotic systems for upper limb rehabilitation are typically tested on stroke patients. Only a fraction of these systems are investigated on subjects suffering from other diseases (see last column of Table 1).

### Type of assistance

The most important terminology introduced in this section is explained in Table 2. Devices for upper limb rehabilitation may provide different types of motion assistance: active, passive, haptic and coaching. **Active devices** provide active motion assistance and possess at least one actuator, thus they are able to produce movement of the upper-extremity. Most of the devices discussed in this survey are active (see Table 1). Such assistance of movements is required if patient is too weak to perform specific exercises. However, even with active devices, an exercise is considered passive when a patient’s effort is not required. For example, devices providing continuous passive motion exercises are active, but those exercises are categorized as passive because the subject remains inactive while the device actively moves the joint through a controlled range of motion. It is not necessary to apply active assistance to resist patient’s movement, to increase patient’s force or to ensure the patient is following the desired trajectory. Instead, **passive devices** may be applied that are equipped with actuators providing resistive force only (i.e. brakes). Such actuators consume less energy and are cheaper than the heavier actuators for active assistance. Devices using only resistive actuators include both devices for physical therapy, e.g. *MEM-MRB*[104] and *PLEMO*[105], and systems for tremor suppression, e.g. *WOTAS*[99] orthosis and a system proposed by Loureiro, et al. [100].

**Table 2:** Glossary of terms concerning type of assistance

**Term**	**Description**
Active device	A device able to move limbs. Under such condition, this device requires active actuators which may increase the weight. It may also apply to subjects completely unable to move their limb.
Passive device	A device unable to move limbs, but may resist the movement when exerted in the wrong direction. This type of device may only be used for rehabilitation of subjects able to move their limbs. It is usually lighter than active device since it possesses no actuators other than brakes.
Haptic device	A device that interfaces with the user through the sense of touch. In most cases it provides some amount of resistive force, often also some other sensation (e.g. vibration). It is sometimes also able to generate specific movements. However, the force it generates is usually small. Haptic devices are commonly used in rehabilitation settings with virtual environments.
Coaching device	A device that neither assists nor resists movement. However, it is able to track the movement and provide feedback related to the performance of the subject. As haptic devices, coaching devices are also commonly used in rehabilitation settings with virtual environments.
Active exercise	An exercise in which subjects actively move their limb, although some assistance of the device may be provided. Such type of the exercise may be performed using any of the above listed types of devices.
Passive exercise	An exercise in which the subject remains passive, while a device moves the limb. This type of exercise requires an active device. Continuous passive motion (CPM) training is an example of passive exercise with active devices.

**Haptic devices** constitute another group of systems interacting with the user through the sense of touch. Haptic devices are similarly classified as either active or passive, depending on their type of actuator. In this report, haptic devices are independently categorized because their main function is not to cause or resist movement but rather to provide tactile sensation to the user. Other non-actuated devices for upper limb rehabilitation do not generate any forces but provide different feedback. These systems are labeled **coaching devices** throughout this report. Because coaching devices are sensorized, they serve as input interface for interaction with therapeutic games in virtual reality (VR) (e.g. *T-WREX*[106], *ArmeoSpring* from Hocoma AG) or for telerehabilitation (i.e. remotely supervised therapy). Coaching systems using video-based motion recognition (e.g. Microsoft Kinect) would also belong to this category if it were not for their lack of any mechanical part in contact with the patient. Therefore, these systems will not be further discussed in this survey.

Passive and non-actuated systems are less complex, safer and cheaper than their active counterparts. However, they are often modified in the development process with more active characteristics. Still, the main characteristic that identifies a non-actuated or passive device is the lack of the ability to perform movement; they may be an option for continuation of the rehabilitation process, rather than for training of people with significant movement disorders at an early stage of rehabilitation.

### Mechanical design

The most important terminology introduced in this section is explained in Table 3. When comparing the mechanical structure of robotic devices for movement rehabilitation often two categories of devices are considered: end-effector-based and exoskeleton-based. The difference between the two categories is how the movement is transferred from the device to the patient’s upper extremity. End-effector-based devices contact the patient’s limb only at its most distal part that is attached to patient’s upper extremity (i.e. end effector). Movements of the end effector change the position of the upper limb to which it is attached. However, segments of the upper extremity create a mechanical chain. Thus, movements of the end effector also indirectly change the position of other segments of the patient’s body as well. Compared to end effectors, exoskeleton-based devices have a mechanical structure that mirrors the skeletal structure of patient’s limb. Therefore movement in the particular joint of the device directly produces a movement of the specific joint of the limb.

**Table 3:** Glossary of terms concerning mechanical design of robots for rehabilitation

**Term**	**Description**
End-effector based device	Contacts a subject’s limb only at its most distal part. It simplifies the structure of the device. However, it may complicate the control of the limb position in cases with multiple possible degrees of freedom.
Exoskeleton-based device	A device with a mechanical structure that mirrors the skeletal structure of the limb, i.e. each segment of the limb associated with a joint movement is attached to the corresponding segment of the device. This design allows independent, concurrent and precise control of movements in a few limb joints. It is, however, more complex than an end-effector based device. Orthoses restricting or assisting movement in one or more joints may be also considered exoskeleton-based devices.
Planar robot	A device, usually end-effector based, moving in a specific plane. Design of planar robots, decreases costs as well as the range of movements that may be exercised. Although this device performs movements in a plane, joints of the limb may still move in a three-dimensional space.
Back-drivability	A property of mechanical design indicating that the patient is able to move the device, even when the device is in passive state. It increases patient safety, because it does not constrain limb movements and keeps patient’s limb in a comfortable position.
Modularity	A property of a device indicating that optional parts may adapt it to a specific condition or simply to perform additional exercises.
Reconfigurability	A property of a device indicating that its mechanical structure may be modified without adding additional parts in order to adapt it to the condition of the subject or to perform other form of training.

The advantage of the **end-effector-based** systems is their simpler structure and thus less complicated control algorithms. However, it is difficult to isolate specific movements of a particular joint because these systems produce complex movements. The manipulator allows up to six unique movements (i.e. 3 rotations and 3 translations). Control of the movements of the patients upper limb is possible only if the sum of possible anatomical movements of patient arm in all assisted joints is limited to six. Increasing the number of defined movements for the same position of the end-point of the manipulator results in redundant configurations of the patient’s arm, thus inducing risk of injuries and complicated control algorithms.

**Figure 1:**
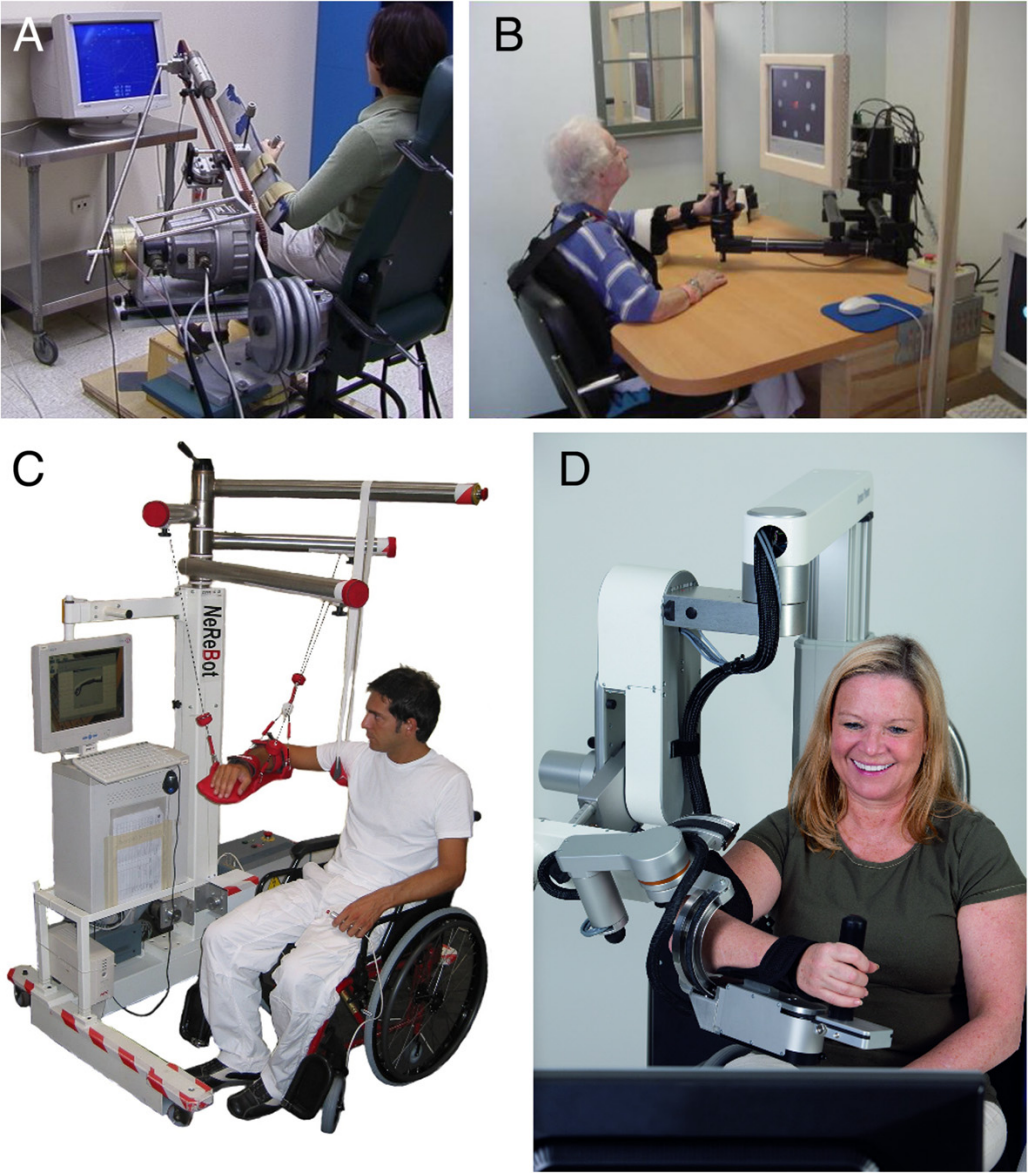
**Examples of mechanical structures of robotic devices for upper limb rehabilitation.A:***ARM Guide* - simple system using linear bearing to modify orientation [136]; **B:***InMotion ARM* - end-effector-based commercial system [133]; **C:***NeReBot* - cable-driven robot, Ⓒ2007 IEEE. Reprinted, with permission, from [111]; **D:***ArmeoPower* - exoskeleton-based commercial system (courtesy of Hocoma AG).

The typical end-effector-based systems include serial manipulators (e.g. *MIT Manus*[107] - Figure 1B, *ACRE*[108]), parallel (e.g. *CRAMER*[109] and a system proposed by Takaiwa and Noritsugu [110], both for wrist rehabilitation), and cable-driven robots (e.g. *NeReBot*[111] - Figure 1C, *MACARM*[112]). The mechanical structure of *HandCARE*[113] may be also recognized as the series of end-effector-based cable-driven robots, each of which induce movement of one finger. In this system a clutch system allows independent movement of each finger using only one actuator.

Application of the **exoskeleton-based** approach allows for independent and concurrent control of particular movement of patient’s arm in many joints, even if the overall number of assisted movements is higher than six. However, in order to avoid patient injury, it is necessary to adjust lengths of particular segments of the manipulator to the lengths of the segments of the patient arm. Therefore setting-up such device for a particular patient, especially if the device has many segments, may take a significant amount of time. Furthermore, the position of the center of rotation of many joints of human body, especially of the shoulder complex [114], may change significantly during movement. Special mechanisms are necessary to ensure patient safety and comfort when an exoskeleton-based robot assists the movements of these joints [114]. For this reason, the mechanical and control algorithm complexity of such devices is usually significantly higher than of the end-effector-based devices. The complexity escalates as the number of DOF increases.

In case of systems for the rehabilitation of the whole limb the number of DOF reaches nine (*ESTEC exoskeleton*[115]) or ten (*IntelliArm*[116]). Some systems for fingers or hand rehabilitation have an even higher number of DOF. Examples include the system proposed by Hasegawa, et al. with eleven DOF [98] and the hand exoskeleton developed at the Technical University (TU) of Berlin with twenty DOF [117]. Even at such a high number of DOF some of these devices still remain wearable (i.e. the user is able to walk within a limited area due to connections to power source and control unit, e.g. *ESTEC* and hand exoskeleton developed at the TU Berlin) or portable (i.e. the area within which the user may walk is not limited, e.g. the system proposed by Hasegawa).

Apart from purely exoskeleton- or end-effector-based devices, there are many **systems combining a few approaches**. In the *ArmeoSpring* system (Hocoma AG) for example, only the distal part – comprising the elbow, forearm and wrist – is designed as an exoskeleton. Therefore the limb posture is statically fully determined (as in exoskeleton-based systems) and the shoulder joint is not constrained, allowing easy individual system adaptation to different patients. A similar concept was applied in Biomimetic Orthosis for the Neurorehabilitation of the Elbow and Shoulder – *BONES*[118]. In that case, a parallel robot consisting of passive sliding rods pivoting with respect to a fixed frame provides shoulder movements. Such application of sliding rods allows internal/external rotation of the arm without any circular bearing element. The distal part allowing for flexing/extending the elbow resembles the exoskeleton structure. In the *MIME-RiceWrist* rehabilitation system [119] the end-effector-based *MIME*[120] system for shoulder and elbow rehabilitation is integrated with the parallel wrist mechanism used in the *MAHI* exoskeleton (known as *RiceWrist*[119]).

Another example is the 6 DOF *Gentle/S*[121] system allowing for relatively large reaching movements (three actuated DOF of the end-effector-based commercial haptic interface *HapticMaster*, Moog in the Netherlands BV [122]) and arbitrary positioning of the hand (connection mechanism with three passive DOF). The *Gentle/S* system was further supplemented with a three-active-DOF hand exoskeleton to allow grasp and release movements. This new nine DOF system is known as *Gentle/G*[123].

The *HEnRiE*[124] is a similar system based on the *Gentle/S* system. In addition to the three active DOF of *HapticMaster*, *HEnRiE* includes a connection mechanism with two passive DOF for positioning of the hand and grasping device (two parallelogram mechanisms allowing parallel opening and closing of fingers attachments) with only one active DOF.

Some systems combine **more than one robot at the same time**. This approach may be considered as the combination of end-effector-approach, where only the most distal parts of robots are attached to the patient’s upper limb, with the exoskeleton-based approach, where movements of few segments are directly controlled at the same time. Use of two robots to control the movements of the limb may allow for mimicking the operations performed by therapist using two hands. Examples of systems using two-robot-concept include *REHAROB*[125] (using two six-DOF manipulators), *iPAM*[126] and *UMH*[127] (both having six DOF in total). Researchers at the University of Twente, in Enschede, Netherlands, made an attempt to use two *HapticMaster* systems to provide coordinated bilateral arm training, but limitations in hardware and software caused the virtual exercise to behave differently to the real-life [128]. In some cases **industrial robots** have also been used. The *REHAROB* uses *IRB 140* and *IRB 1400H* from ABB Ltd., while *MIME*[120] uses *PUMA 560* robot. In general, industrial robots reduce costs; however, such robots have significantly higher impedance than the human upper limb and, according to Krebs, et al. [129], should not be in close physical contact with patients. Therefore most of the robots used for the rehabilitation of the upper limb are designed with a low intrinsic impedance. Some of those devices are also **back drivable** (e.g. *HWARD*[130] and *RehabExos*[131]), meaning that the patient’s force is able to cause movement of those devices when they are in passive state. Back-drivability further increases safety of the patient because the device does not constrain patient movements. It also allows for using the device as an assessment tool to measure patient’s range of motion.

The majority of the devices presented in Table 1 allow movements in three dimensions; however there are also **planar robots**, i.e., systems allowing movements only on a specified plane (e.g. *MEMOS*[132] and *PLEMO*[105]). Also the *MIT Manus* system initially allowed movements only on one plane [107]. Subsequently, an anti-gravity module added possibility to perform vertical movements [133] (Figure 1B). Designing the device as a planar robot reduces the range of movements that can be exercised for particular joint. It also reduces the cost of the device. Furthermore, when the working plane is well selected, the range of training motion may suffice in most of therapeutic scenarios. Some of such planar devices allow changes in the working space between horizontal and vertical (*Braccio di Ferro*[134]) or even almost freely selecting the working plane (e.g. *PLEMO* and *Hybrid-PLEMO*[135]). It further increases the range of possible exercise scenarios while keeping the cost of the device at a minimum.

In the *ARM Guide*[136] (Figure 1A) and *ARC-MIME*[137] systems, with which patients practice reaching movements, the working space is limited to linear movements because the forearm typically follows a straight-line trajectory. However the orientation of the slide that assists forearm movements can be adjusted to reach multiple workspace regions and fit different scenarios.

**Modularity** and **reconfigurability** are concepts that could reduce therapy costs by adopting therapeutic devices for various disabilities or stages in patient recovery. However there are still only a few systems using these concepts. For example, *InMotion ARM* robot (the commercial version of *MIT Manus*, previously called *InMotion 2.0*) from Interactive Motion Technologies, Inc., may be extended by *InMotion WRIST* robot (previously *InMotion 3.0*), developed at MIT [138] as a stand-alone system, and *InMotion HAND* add-on module (previously *InMotion 5.0*) for grasp and release training. Another example of modular system is *MUNDUS*[101], consisting of various modules that may be included depending on the patient condition, starting from muscle weakness to complete lost of residual muscle function. For example as command input residual voluntary muscular activation, head/eye motion, or brain signals may be used. However, this system’s complexity might make commercialization of the device very difficult.

**Figure 2:**
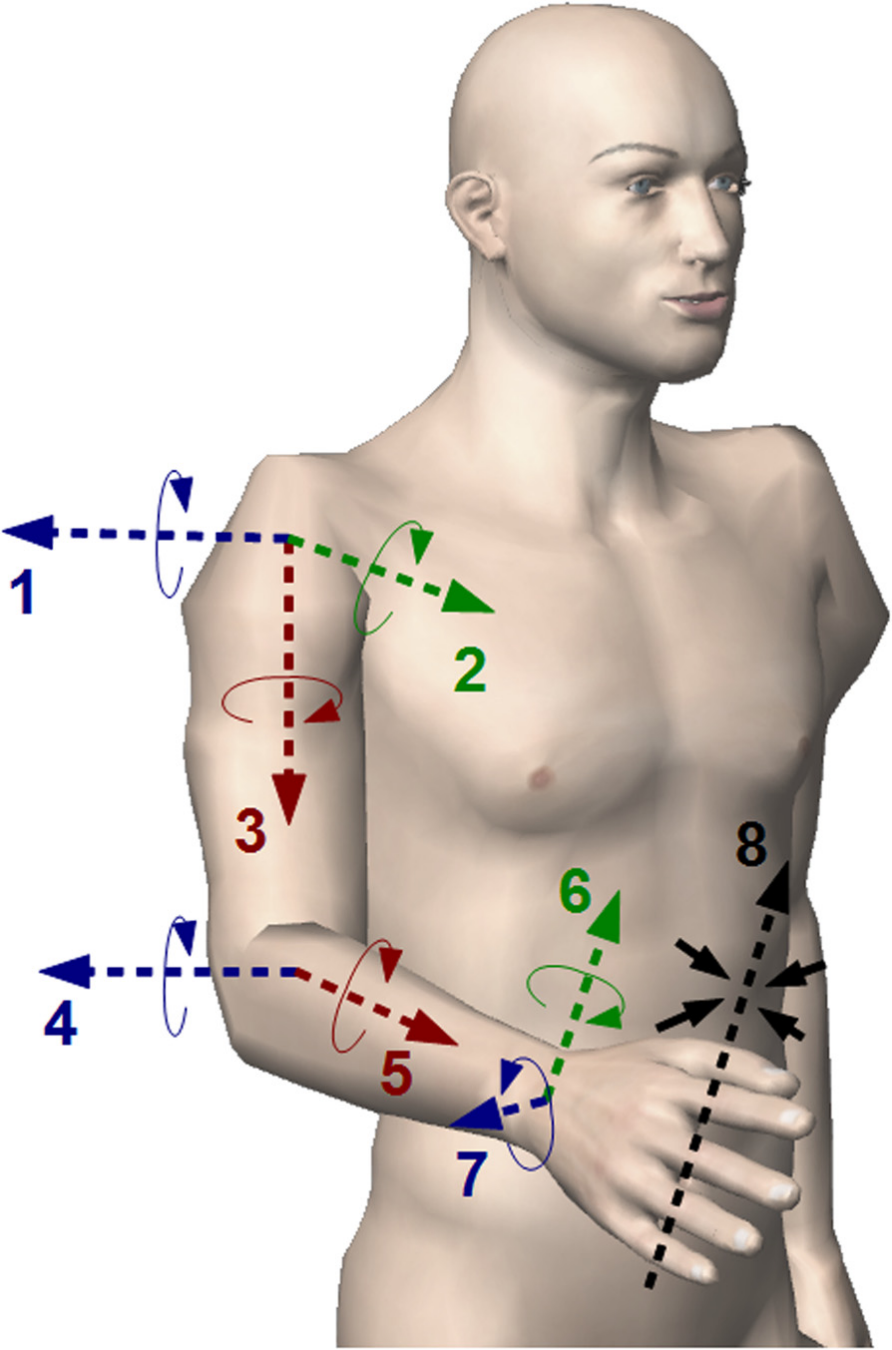
**Main movements (degrees of freedom) of the upper extremity.** 1: arm flexion/extension; 2: arm adduction/abduction; 3: arm internal(medial)/external(lateral) rotation; 4: elbow flexion/extension; 5: forearm pronation/supination; 6: wrist flexion/extension; 7: wrist adduction(ulnar deviation)/abduction(radial deviation); 8: hand grasp/release.

A very interesting solution was implemented in the *Universal Haptic Drive (UHD)*[139]. It has only two DOF and, depending on the chosen configuration, it can train either shoulder and elbow during reaching tasks or forearm (flexion/extension) and wrist. For the latter setting option, it is also possible to select a flexion/extension or pronation/supination training for the wrist. See Figure 2 for an explanation of anatomical terms used for description of upper limb motion.

### Actuation and power transmission

The most important terminology introduced in this section is explained in Table 4. Traditionally, energy to the actuators is provided in three forms: electric current, hydraulic fluid or pneumatic pressure. The selection of the energy source determines the type of actuators used in the system. Most of the devices for upper extremity rehabilitation use electric actuators but there are also other systems with pneumatic and hydraulic actuators. The **electric actuators** are most common because of their ease to provide and store electrical energy as well as their relatively higher power. Various types and sizes of electrical motors and servomotors are currently commercially available. Some authors (e.g. Caldwell and Tsagarakis [140]) argue that electric actuators are too heavy, compared to their pneumatic counterparts, and their impedance is too high to be used in rehabilitation applications. However, the relatively high power-to-weight ratio of pneumatic actuators is achieved by neglecting the weight of the power source. Adding an elastic element in series with the actuator may also mitigate the high impedance of electric motors. This concept lead to the development of the so called **Series Elastic Actuators (SEAs)**. SEAs decrease inertia and user interface impedance to provide an accurate and stable force control [141], thus increasing the safety of the patient. The disadvantage of application with an elastic element is the lower functional bandwidth. Still, the field of rehabilitation does not usually require high bandwidths. SEAs with electric motors are investigated in *MARIONET*[142] and *UHD*[139] systems, as well as in systems proposed by Vanderniepen, et al. (referred to as MACCEPA actuators) [143] and Rosati, et al. [144].

**Table 4:** Glossary of terms concerning actuation of robots for rehabilitation

**Term**	**Description**
Electric actuators	Actuators powered by electric current. They are the most common because they easily provide a relatively high power and are able to store energy. There is a wide selection of commercially available electric actuators; however, some of them are heavy and/or their impedance is too high for rehabilitation settings.
Hydraulic actuators	Actuators powered by hydraulic pressure (usually oil). They are able to generate high forces. Their system is relatively complex considering the maintenance of pressurized oil under pressure to prevent leakage. Commercial hydraulic actuators are also heavy, therefore, only specially designed hydraulic actuators are used in rehabilitation robotics.
Pneumatic actuators	Actuators powered by compressed air. They have lower impedance and weigh less than electric actuators. Special compressors or containers with compressed air are required for power.
Pneumatic Artificial Muscle (PAM, McKibben type actuator)	A special type of pneumatic actuator with an internal bladder surrounded by a braided mesh shell with flexible, but non-extensible threads. Because of their specific design, an actuator under pressure shortens, similarly to the contracting muscle. It is relatively light and exerts force in a single direction. It is difficult to control because of its slow and non-linear dynamic functions.
Series Elastic Actuator (SEA)	A generic name used for a mechanism with an elastic element placed in series with an actuator. This solution is relatively often met in the design of rehabilitation robots. It decreases the inertia and intrinsic impedance of the actuator to allow a more accurate and stable force control and increase patient safety.
Functional Electrical Stimulation (FES)	It is a technique that uses electrical current to activate nerves and contract their innervated muscles. It produces the movement of the limb using natural actuators of the body. However, it is difficult to achieve precise and repeatable movement using this technique and it may be painful for the patient.

A few systems use **pneumatic actuators**. Pneumatic actuators are lighter and have lower inherent impedance than the electric counterparts. Because such actuators require pneumatic pressure, the system is generally either stationary (e.g. *Pneu-WREX*[145]), its service area is limited (e.g. *ASSIST*[146]) or the compressor is installed on the patient’s wheelchair (e.g. system proposed by Lucas, et al. [147]). Special type of pneumatic actuators, called **Pneumatic Artificial Muscles (PAMs)**, Pneumatic Muscle Actuators or McKibben type actuators are often used in rehabilitation robotics (e.g. *Salford Arm Rehabilitation Exoskeleton*[148] or system proposed by Kobayashi and Nozaki [149]). Such actuators consist of an internal bladder surrounded by braided mesh shell with flexible, but non-extensible, threads. When the bladder is pressurized, the actuator increases its diameter and shortens according to its volume, thus providing tension at its ends [150]. Due to such physical configuration, PAMs’ weight is generally light compared to other actuators, but also have slow and non-linear dynamic response (especially large PAMs), in consequence they are not practical for clinical rehabilitation scenarios [131,151]. In addition, at least two actuators are necessary in order to provide antagonistic movements due to the unidirectional contracting. The *ASSIST* system has a special type of PAM with rotary pneumatic actuators that allows bending movements [146].

A total of four systems using **hydraulic actuators** were identified in this survey. All four of them are not standard and use actuators developed specially for that purpose. Reasons to evade industrial hydraulic actuators include weight, impedance, fluid leakages and difficulties to provide fluid. Large, noisy systems are usually necessary for that purpose. Mono-and bi-articular types of Hydraulic Bilateral Servo Actuators (HBSAs) are used in the wheelchair-mounted exoskeleton proposed by Umemura, et al. [152]. Miniaturized and flexible fluidic actuators (FFA) were applied in the elbow orthosis proposed by Pylatiuk, et al. [153]. Hydraulic SEAs are used in two other systems: the *Dampace* system [154] is equipped with powered hydraulic disk brakes; the *Limpact* system [155], developed by the same group, uses an active rotational Hydro-Elastic Actuator (rHEA).

In passive systems, it is often desired to modify the amount of resistance during the exercise. This modification increases the resistance when the patient departs from the desired trajectory or to provide haptic feedback for VR interactions. In existing systems, different solutions for provision of **adjustable resistive force** have been implemented. Powered hydraulic brakes, for example, controlled by electromotors in a SEA are used in *Dampace* system [154]. Magnetic particle brakes are used in *ARM Guide*[136] (Figure 1A), in its successor *ARC-MIME*[137] to resist other than longitudinal movements of the forearm, and in the device for training of multi-finger twist motion proposed by Scherer, et al. [156]. A few groups have also investigated the application of brakes incorporating magnetorheological (MRF brakes) and electrorheological fluids (ERF brakes). These fluids change their rheological properties (i.e. viscosity) depending on the applied magnetic or electric field, respectively. Thanks to those properties it is possible to achieve brakes with high-performance (with rapid and repeatable brake torque) [105]. MRF brakes are used in *MRAGES*[157] and *MEM-MRB*[104] systems. ERF brakes are used in *PLEMO*[105] and *MR_CHIROD v.2*[158] systems. The same group that developed the *PLEMO* also proposed ERF clutches to control the force provided by an electric motor in active systems. Such an actuation system was implemented in *EMUL*[159], *Robotherapist*[160] and *Hybrid-PLEMO*[135] devices.

The natural actuators of body muscles can be used instead of external actuators. For this purpose, an electrical stimulation of the muscles leading to their contraction can be applied. This specific electrical stimulation is known as **Functional Electrical Stimulation (FES)**. FES significantly reduces the weight of the device. From a therapeutic point of view, FES allows patients to exercise muscles, improving muscle bulk and strength and preventing muscular atrophy [161]. It has been also shown that FES, complemented by conventional physiotherapy, may enhance the rehabilitation outcome [162]. However, FES may cause strong involuntary muscle contractions and can be painful for patient. Furthermore, it is difficult to control movements using FES because of the non-linear force characteristic of contracting muscles, muscles fatigue and dependency of the achieved contraction on the quality of the contact between stimulating electrodes and the body tissue. There are two commercial systems using FES for upper limb rehabilitation: *Ness H200* (Bioness, Inc., US) and *NeuroMove* (Zynex Medical, Inc., US). Two other systems combining FES with assistive force were proposed by Freeman, et al. [163] and Li, et al. [164].

When selecting actuators, it is also important to consider their location, especially with exoskeleton-based mechanical structures. The actuators can be placed distally, close to the joints on which they actuate (e.g. *ArmeoPower* system, Figure 1D). This specification simplifies the power transmission by using direct drives. However, it increases the weight of the distal part of the device and inertia and makes it more difficult to control the system. On the other hand, locating the actuators in the proximal part of the device, often in the part that remains constrained, reduces the weight and inertia of the distal part. However, a power transmission mechanism complicates the mechanical structure and may lead to difficulties with control due to friction. For example, the same group who developed *InMotion HAND* system proposed an earlier prototype of the hand module with eight active DOF and cable-driven mechanism for power transmission. The friction in that mechanism and its level of complexity was too high for clinical scenarios [165]. Nevertheless, there are systems, in which power transmission using cables and gear drives was successfully applied, like for example *CADEN-7*[97] and *SUEFUL-7*[166].

### Control signals

The most important terminology introduced in this section is explained in Table 5. Various signals may be used as control input of the device. **Switches** are often used to simplify design. Examples include the *PowerGrip* system from Broaden Horizons, Inc., hand held triggers (e.g. FES based system for grasp assistance proposed by Nathan, et al. [167]) and a joystick (e.g.*MULOS*[168]). Most of the systems having more complex control strategies use either **kinematic, dynamic or a mix of both input signals** (see Table 1 for a comparison). The type of the signal used as control input is partially determined by the low-level control strategy and vice-versa. In some cases, signals provided by actuator encoders (concerning position or torque) may be directly used for control purposes. However, usually torque measured by the encoder is a sum of the torque exerted by the user on device and internal torques in the device. Therefore, for better control of forces between patient and device, it is useful to apply additional sensors that will measure those forces directly.

**Table 5:** Glossary of terms concerning input control signals of robots for rehabilitation

**Term**	**Description**
Dynamic signals	Signals related to the torque or force exerted by the subject on various joints of the device (exoskeleton-based device) or at its end effector (end-effector-based device).
Kinematic signals	Signals related to positions, orientations, velocities and accelerations of particular segments or joints of the device or of the limb.
Trigger signal	A signal initiating a specific action. In simple cases, a switch or a button triggers the signal. In more complex cases, a threshold value of some signal is specified to trigger the action (e.g. a sEMG value corresponding to a level of muscle contraction).

Some systems use **surface electromyography (sEMG)** as an input signal, which provides information about intention of the person to perform particular movement. Therefore it is possible to detect and support it. Most of such systems support elbow movements, as sEMG signals from muscles controlling this joints (i.e. biceps brachii or triceps brachii) are relatively easily measured. Among proposed solutions are both stationary (e.g. systems proposed by Rosen, et al. [169] and Kiguchi, et al. [170]) and portable systems (e.g. systems proposed by Ögce and Özyalçin [171] and Pylatiuk, et al. [153]). So far the most successful of those systems is the one DOF portable orthosis developed at the Massachusetts Institute of Technology (MIT), Cambridge, US [172]. The system successfully sustained clinical trials, received FDA approval and was commercialized as *Myomo e100* system (Myomo, Inc.) [173]. Examples of sEMG-controlled systems supporting movements of other joints include those proposed by Kiguchi, et al. [114] for shoulder rehabilitation, *W-EXOS*[174] for forearm and wrist rehabilitation, *PolyJbot*[175] for wrist rehabilitation, *SUEFUL-7*[166] exoskeleton for whole limb (excluding fingers) movement assistance, *TU Berlin Hand Exoskeleton*[117] for fingers rehabilitation, as well as 11-DOF portable orthosis for grasp assistance proposed by Hasegawa, et al. [98]. The sEMG signals from the contralateral healthy limb have been also used to control movements of the affected one (see system proposed by Li, et al. [176]). The concept of using **movements of the not affected limb** to control motion of the affected one has been also implemented in *Bi-Manu-Track* system (Reha-Stim, Germany), *ARMOR* exoskeleton [177] and device proposed by Kawasaki, et al. [178]. Using the other limb to control the affected one is especially useful during rehabilitation after stroke. In cases of hemiparesis (or hemiplegia), often only one side of the body is affected.

In some systems also **contact-less movement detection** methods have been used. For example, Ding, et al. [179] proposed a system to assist the load of arbitrary selected muscles using motion capture systems in order to calculate the actual muscle force.

### Feedback to the user

Different types of feedback may be provided to the user, among them: visual, tactile, audio and in the form of electrical stimulation. Some systems, for example those proposed by Lam, et al. [180] and Nathan, et al. [167], use vibrational stimulation of the muscle tendons to support their contraction. It was also suggested that providing tactile feedback to flexor and extensor surfaces of the skin at the appropriate location could produce more naturalistic movements and improve clinical outcomes [3]. Some other systems combine other types of the feedback, for example system proposed by Casellato, et al. [181] combines visual and haptic feedback to improve motor performance of children with dystonia.

A significant number of systems provide **training in virtual reality (VR) scenarios**. VR provides a much more interesting training surrounding to the patient, compared to the typically available conditions presented in therapeutic units. Furthermore, it allows for fast modification of training scenarios, increasing patient attention and motivation to perform the exercise. Therefore it may also improve positive outcome of the therapy. It also adapts the system for various patients in a very short time frame and restarts the task if the object was dropped or misplaced. Haptic devices are especially well suited for provision of therapy in VR because they provide an impression of manipulating the virtual objects. Some groups developed own versions of haptic systems. For example Takahashi, et al. [182] proposed haptic device for arm rehabilitation, which can apply multiple types of force including resistance, assistance, elasticity, viscosity and friction. Other examples are: a two DOF *Haptic Interface for Finger Exercise* (*HAFI*), which provides rehabilitation of only one finger at a time [183]; a force reflecting glove, named *MRAGES*, using magneto-rheological fluid [157]; *MR_CHIROD v.2*, a one DOF grasp exercise device for functional magnetic resonance imaging [158] and force-feedback glove *Rutgers Master II-ND*[184], developed at the Rutgers University (Piscataway, US) and used in hand therapy scenarios (e.g. [185-187]).

Many groups have investigated application of a few of commercial haptic devices for rehabilitation of upper extremity. Among such haptic interfaces are: - *HapticMaster* incorporated for example in *Gentle/S*[121] (for other examples see Table 1),- in-parallel robots *Phantom Omni* and *Premium* (Geomagic, Inc., US) - used e.g. in experiments performed by Casellato, et al. [181], Brewer, et al. [188], and Xydas and Louca [189], - parallel robot *Falcon* (Novint Technology, Inc., US) - used in *My Scrivener* system for hand writing training (Obslap Research LLC, US) [190],- force-feedback glove *CyberGrasp* (CyberGlove Systems LLC,US) - used among others in therapeutic scenarios investigated by Adamovich, et al. [191,192].

Because the entertainment industry have recently introduced many new devices to capture motion of the healthy people for interaction with VR-based games, it may be expected that soon some of those devices will be also adapted for rehabilitation purposes, providing so called “serious games”.

### Control strategy

The most important terminology introduced in this section is explained in Table 6. Following the example of Marchal-Crespo and Reinkensmeyer [193] we will consider “high-” and “low-level” control strategies used by rehabilitation robots. “High-level” control algorithms are explicitly designed to provoke motor plasticity whereas “low-level” strategies control the force, position, impedance or admittance factors of the “high-level” control strategies.

**Table 6:** Glossary of terms concerning control strategy of robots for rehabilitation

**Term**	**Description**
“High-level” control strategy	A control strategy with control algorithms explicitly designed to induce motor plasticity.
Assistive control	A “high-level” control strategy in which a device provides the physical assistance to aid the patient in accomplishing an intended movement.
Challenge-based control	A “high-level” control strategy in which a device challenges the patient to accomplish an intended movement.
Haptic stimulation	A “high-level” control strategy in which a robotic device is used as a haptic interface to perform activities in virtual reality environment.
Couching control	A “high-level” control strategy in which a robotic device neither physically assists nor resists the movement of the subject. It only quantifies and provides feedback (visual, acoustic or other) concerning the performance of the subject during exercise.
“Low-level” control strategy	A control strategy considered in the implementation of the “high-level” control strategy in a device by appropriate control of the force, position, impedance or admittance.
Admittance control	A “low-level” control strategy in which the force exerted by the user is measured, and the device generates the corresponding displacement.
Impedance control	A “low-level” control strategy in which the motion of the limb is measured and the robot provides the corresponding force feedback.

This terminology is mostly based on the one proposed by Marchal-Crespo and Reinkensmeyer [193].

#### “High-level” control strategies

There is a myriad of “high-level” control strategies for robotic movement training. This section briefly summarizes the classification of those strategies presented by Marchal-Crespo and Reinkensmeyer [193]. They identify four categorizes of control strategies: (a) assistive control, (b) challenge-based control, (c) haptic stimulation, and (d) non-contacting coaching. Although some systems may fall into a few of these categorizes, this classification well illustrates main notions in the “high-level” control of robotic devices for upper limb rehabilitation. Those control strategies in most cases correspond also to active, passive, haptic and coaching types of motion assistance described before.

The **assistive control strategy** makes tasks safer and easier to accomplish, allowing more repetitions. There are four types of assistive strategies: impedance-based, counterbalance-based, EMG-based and performance-based adaptive control. In the impedance-based strategy, the patient follows a particular trajectory. The device does not intervene as long as the patient follows this trajectory. However, as the patient leaves the trajectory, the device produces a restoring force that increases along with the deviation from the desired trajectory. Often some margin of deviation from allowed trajectory is accepted before restoring force is provided. Counterbalance-based strategies provide a partial, passive or active weight counterbalance to a limb, those making the exercises easier, as the amount of force needed to move the limb against the gravity may be significantly reduced. EMG-based approach uses the patient’s own sEMG signals to either trigger or proportionally control the assistance. Both of those approaches encourage patients’ effort. However, the triggered method is more susceptible to slacking, as the patient may learn to provide only the amount of force needed to trigger the assistance. Finally, the performance-based adaptive control strategies monitor the performance of the patient and adapt some aspects of the assistance (e.g. force, stiffness, time, path) according to the current performance of the patient, as well as performance during particular number of preceding task repetitions.

##### Challenge-based control strategies

fall into three groups: resistive, amplifying error and constraint-induced. The resistive strategies resist the desired movements, those increasing the effort and attention of the patient. The error amplifying strategies are based on the theory that faster improvements are achieved when error is increased [194]. Therefore they track the deviations from the desired trajectories and either increase the observed kinematic error or amplify its visual representation on the screen. The constraint-induced robotic rehabilitation strategy, similarly to conventional constraint-induced therapy, promotes the use of the affected limb by constraining the not affected one.

##### Haptic stimulation strategies

make use of haptic devices described above, providing tactile sensation for interactions with virtual reality objects. These strategies support training of basic ADLs in safe conditions and without long set-up. They provide alternate tasks in various environments, attracting attention and providing conditions for implicit learning.

##### Non-contacting coaching strategy

is applied in systems that do not contact participants but rather monitor their activity and provide instructions to the patient. Instructions indicate how to perform particular activities or what should be improved. Since such devices do not contact the patient, they are not applicable for systems described herein. However, this category may be extended to include also some sensorized, but not-actuated exoskeletons, such as the gravity balancing orthosis *T-WREX*[106].

#### “Low-level” control strategies

Different “low-level” control strategies are combined to develop “high-level” rehabilitation strategies. Many “low-level” control strategies may be proposed during following stages of development of a robotic rehabilitation device. This report provides a short description of basic approaches and does not include a comprehensive comparison of “low-level” control strategies. General books on control engineering provide a more detailed description, in addition to articles referenced in Table 1.

As the rehabilitation robots interact with human body, it is necessary to consider the manipulator and patient as a coupled mechanical system. The application of force or position control is not enough to ensure appropriate and safe dynamic interaction between human and robot [195]. Other control strategies, such as impedance or admittance control are implemented in most of the robots for upper limb rehabilitation. In the **impedance control approach** the motion of the limb is measured and the robot provides the corresponding force feedback, whereas in the **admittance control approach** the force exerted by the user is measured, and the device generates the corresponding displacement. The advantages and disadvantages of the impedance and admittance control systems are complementary [196]. In general, robots with impedance control have stable interaction but poor accuracy in free-space due to friction. This low accuracy can be improved using inner loop torque sensors and low-friction joints or direct drives. Admittance control in contrast compensates the mass and friction of the device and provides higher accuracy in non-contact tasks, but can be unstable during dynamic interactions. This problem is eliminated using SEAs. Devices using admittance control require also high transmission ratios (e.g. harmonic drives) for precise motion control [196]. In some cases both of theses approaches may be combined together. Impedance control strategy has been implemented for example in *MIT Manus*[107] (Figure 1B) and *L-Exos* exoskeleton [197], admittance control is found in *MEMOS*[132] and *iPAM*[126].

### Clinical evidence

#### Clinical studies

As previously discussed, there has been a significant effort during last two decades to improve the design and control strategy of robotic rehabilitation devices. Yet, less has been done to prove the efficacy of such systems in rehabilitation settings. Although the results of clinical evaluation of therapy applying robots are still sparse, the problem is slowly being recognized. The focus in rehabilitation robotics is starting to move from technical laboratories to clinics. References to clinical trials in which robotic rehabilitation devices have been used are provided in the last column of Table 1. The classification of clinical trials used in this review is summarized in Table 7.

**Table 7:** The classification of clinical trials of rehabilitation robots used in this review

**Term**	**Description**
Category 0	Initial feasibility studies: Trials performed with low number of healthy volunteers, often using the prototype of a device, in order to evaluate its safety and clinical feasibility.
Category I	Pilot Consideration-of-Concept studies: Clinical trials aimed at testing device safety, clinical feasibility and potential benefit. They are performed in a small population of subjects suffering from the target disease. There is either no control group in the trial, or healthy subjects are used as control.
Category II	Development-of-Concept studies: Clinical studies aiming at verification of device efficacy. Include a standardized description of the intervention, a control group, randomization and blinded outcome assessment.
Category III/IV	Demonstration-of-Concept-Studies/Proof-of-Concept studies: Further evaluation of the device efficacy. Similar to category II, however, usually these are multicentered studies with high number of participants.

This classification is based on guidelines provided by Lo [198] and supplemented by Category 0.

From the developer and manufacturer’s point of view, there may be at least three objectives in performing clinical trials. The first one is to address regulatory requirements. The devices described in this review are considered medical devices in most countries and as such the studies proving device efficacy and safety may be required before they are authorized for distribution. Although in some cases the exemption from providing the clinical data may be granted, e.g. if the device is recognized as low risk (Class 1 device in the European Union and the USA) or if equivalent device has been already approved for commercialization, the clinical data may be required by health insurance authorities in order to provide reimbursement. In this case the objective of the trial is to obtain a proof of clinical or financial benefit of the use of the device as compared to the existing modes of therapy. The third objective of clinical trials is to provide the professional community with the clinical evidence of device’s efficacy. Although, the three objectives may seem to be similar, the requirements are not the same, therefore when designing a clinical trial it should be considered if the obtained results will allow to satisfy requirements of those three objectives. For the study design requirements to satisfy the marketing and reimbursement objectives, we refer the readers to the legal regulations in the country of interest. Whereas, for a review on the process to design a clinical train with sound scientific results we refer to Lo [198].

From the clinical point of view, the objective of the clinical study may be different than to validate a particular device. For therapists the robotic device is a tool that provides a therapy protocol rather than an end product [198], thus they are rather interested in responses to questions concerning optimal training intensity, disorders for which such form of training may be beneficial, and whether robotic therapy should substitute or complement other forms of therapy.

**Figure 3:**
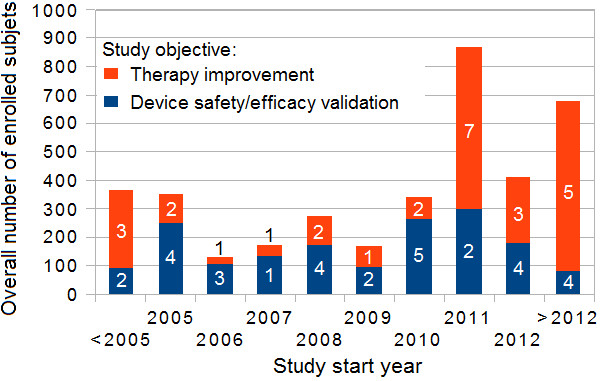
**Number of clinical studies and enrolled subjects depending on study objective and start year.** Results based on a search in the clinicaltrials.gov database in October 2013. Numbers on and above the bars indicate the number of studies in each category starting in the particular year.

This survey includes a search into the US Clinical Trials database (http://clinicaltrials.gov/) from October 2013 using a combination of keywords: robotic, robot, therapy and rehabilitation. This is an web-based database existing since 1997 and maintained by the US National Library of Medicine at the National Institutes of Health. Under the American Food and Drug Administration Amendments Act (FDAAA) of 2007 all the applicable clinical trials (what concerns category II and III/IV studies in our survey) performed in the USA and starting after 2007 have to be registered in this database. However, it includes also some category I studies and many other studies performed in other countries. Results of this search identified 197 clinical trials out of which 62 are relevant to this survey. The selected trials are divided into two categories. The main objective of the first category is to proof the efficacy or safety of the device, therefore there was either no control group or a control group was undergoing the standard form of the therapy. The main objective of the second category is to determine a more efficient form of the therapy. In the latter category, the participants were assigned to groups undergoing similar forms of therapy, but at different intensities, using various devices or undergoing various forms of therapy in different order. A total of 31 studies aim at device safety or efficacy validation while 27 address better forms of therapy. A total of four trials were excluded. The objective of these trials was to validate other forms of therapy; devices described in this review have only been used as a reference. As indicated in Figure 3, the number of participants enrolled in studies for therapy improvement significantly increased during last three years compared to the number of participants in the device safety/efficacy validation studies. This suggests that the objective of the studies changes from validating forms of therapy to finding optimal applications methods. This survey identified a total of 21 devices out of the 58 clinical studies. However, it was not possible to determine the robotic device in 11 studies. Surprisingly, almost only stroke survivors (54 studies) were enrolled. In the remaining four studies subjects with cerebral palsy, spinal cord injury, traumatic brain injury and rotator cuff tear were involved.

#### Outcomes of clinical studies

Many questions concerning effective robotic upper-limb rehabilitation still remain unanswered. One of the most important reasons is that the most effective interventions to optimize neural plasticity are still not clear and it is not possible to implement them in rehabilitation robotics [7]. The other is that the results of the clinical controlled trials remains limited and those already available are difficult to compare with each other [7,193]. It is also questionable which measures should be used to evaluate the effects of therapy and which outcome should be compared: short-term or long-term. Scales based on evaluation of abilities influencing the quality of life are often not objective enough, since they rely on therapist expertise.

Although it is not possible to indicate the best control strategy for the rehabilitation, there is already some evidence showing that some strategies are producing better outcomes, whereas some can even decrease recovery time compared to possible non-robotic strategies [193].

##### Most accepted theories about robotic rehabilitation

are clear: The goal of the rehabilitation training is not only to maximize the number of repetition but to maximize the patients attention and effort as well [3]. The monotonous exercises provide worse retention of a skill compared with alternate training [7]. Adaptive therapy and assistance as needed provide better results as fixed pattern therapy [193]. Robotic therapy can possibly decrease recovery if it encourages slacking since the patient may decrease effort and attention due to the use of adaptive algorithm [193]. Because learning is error based, faster improvement may be achieved when error is increased [194]. Implicit learning, allowing patients to learn skills without awareness, may result in greater learning effect [7]. Many functional gains are more dependent on wrist and hand movements than on the mobility of shoulder and elbow [7]. It is not the maximal voluntary contraction (strength of the muscles) but appropriately timed activity of agonist and antagonist (coordination of the movements) that significantly improve the rehabilitation [3].

As previously stated, the objective of this report is not to review the results of clinical studies performed so far. A detailed review of clinical studies is referenced in other publications [7,198-202]. The most important results are still worth mentioning. Systematic review and meta-analysis of the trials performed in stroke patients suggest that robotic training improves motor impairment and strength but do not improve ability to perform ADLs [199,200]. The results of the first large randomized multicenter study in which training with *MIT-Manus* robotic system have been compared with intensive therapist-provided therapy and usual care have revealed that there is no significant difference in the outcomes of the two intensive forms of the therapy [203]. Thus the most important advantage of robotic systems is their ability to provide intensive repetitive training without over-burdening therapists [204]. Another advantage is the ability to provide more motivating training context, by means of a computer gaming environment with quantitative feedback to motivate practice [205]. Concerning cost-effectiveness of robotic rehabilitation, the results of the previously mentioned multicenter trial have shown that when the total cost of the therapy is compared, i.e. the cost of the therapy plus the cost of all the other health care use, the costs of the two forms of the intensive therapy (i.e. robot-assisted and therapist-provided) are similar [203]. However, the cost of technology is expected to decrease, as opposed to the cost of human labor. Therefore cost-effective advantage toward robot-therapy may be expected [198].

## Conclusions

Due to population changes, shortage of professional therapists, and the increasing scientific and technical potential, many research groups have proposed devices with the potential to facilitate the rehabilitation process. Many devices for upper limb rehabilitation have already been proposed. A vast majority of these proposed devices are technically advanced and are designed for clinical settings. However, there is still significant need to improve efficiency and reduce cost of home-based devices for therapy and ADLs assistance. The effectiveness of robotic over conventional therapy is arguable and the best therapy strategy is still not clear. The situation may change soon, because more and more devices are being commercialized and more scientific results will be available. It may encourage next groups to propose their own solutions. Developing new devices and improving those already in the market will be easier, when taking advantage from the already existing solutions. We hope that this survey will help to navigate between those solutions and select best of them, thus facilitating development of new and better systems for robotic upper limb rehabilitation. We also hope that it will be a valuable source of information for all the professionals looking for a comprehensive reference.

## Competing interests

The authors declare that they have no competing interests.

## Authors’ contributions

PM performed systematical review of papers and drafted the manuscript. JE, KGH, and AJ contributed to the review of various systems and revised the manuscript. SL contributed to the concept, paper structure and revised the manuscript. All authors read and approved the manuscript.
